# Buccal dental-microwear and dietary ecology in a free-ranging population of mandrills (*Mandrillus sphinx*) from southern Gabon

**DOI:** 10.1371/journal.pone.0186870

**Published:** 2017-10-26

**Authors:** Alice M. Percher, Alejandro Romero, Jordi Galbany, Gontran Nsi Akoue, Alejandro Pérez-Pérez, Marie J. E. Charpentier

**Affiliations:** 1 Institut des Sciences de l’Évolution de Montpellier (ISE-M) UMR5554, Univ. Montpellier, CNRS, IRD, EPHE, Montpellier, France; 2 Departamento de Biotecnología, Universidad de Alicante, Alicante, Spain; 3 Center for the Advanced Study of Human Paleobiology, Department of Anthropology, The George Washington University, Washington, DC, United States of America; 4 Université des Sciences et Techniques de Masuku, Franceville, Gabon; 5 Secció de Zoologia i Antropologia, Departament de Biologia Evolutiva, Ecologia i Ciències Ambientals, Facultat de Biologia, Universitat de Barcelona, Barcelona, Spain; Max Planck Institute for Evolutionary Anthropology, GERMANY

## Abstract

Analyses of dental micro- and macro-wear offer valuable information about dietary adaptations. The buccal surface of the teeth does not undergo attrition, indicating that dental microwear may directly inform about food properties. Only a few studies have, however, investigated the environmental and individual factors involved in the formation of such microwear in wild animals. Here, we examine variation of buccal microwear patterns of mandibular molars in a large free-ranging population of mandrills (*Mandrillus sphinx*). We first explore the influence of seasonality and individual’s sex, age and tooth macrowear–expressed as the percent of dentine exposure (PDE)–on six microwear variables. Second, we analyze the interplay between individual’s diet and PDE. In a last analysis, we revisit our results on mandrills in the light of other primate’s microwear studies. We show that the average buccal scratch length and the frequency of vertical buccal scratches are both higher during the long dry season compared to the long rainy season, while we observe the inverse relationship for disto-mesial scratches. In addition, females present more disto-mesial scratches than males and older individuals present higher scratch density, a greater proportion of horizontal scratches but a lower proportion of vertical scratches than young animals. PDE yields similar results than individual’s age confirming earlier results in this population on the relationship between age and tooth macrowear. Because seasonality and individual characteristics are both known to impact mandrills’ diet in the study population, our results suggest that buccal microwear patterns may inform about individual feeding strategies. Furthermore, PDE increases with the consumption of potentially abrasive monocotyledonous plants, independently of the individuals’ age, although it is not affected by food mechanical properties. Finally, buccal scratch densities by orientation appear as relevant proxies for discriminating between different primate taxa.

## Introduction

Teeth are at the interface between the internal milieu of an organism and its environment; they experience accumulation and erasure of traces continuously over a lifetime. These traces, or dental microwear, result from interactions between dental tissue and the external environment, including food items and the extrinsic abrasive particles that cover them [1]. Dental microwear analysis appears therefore as a useful tool to identify the physical properties (e.g., abrasiveness, hardness) of the food items ingested and may help to reconstruct the diet of extinct and extant animal species [2–6]. For example, 3D textural analysis of dental microwear of extinct ruminants help to discriminate grass feeders from browse feeders [7,8]. Moreover, both 2D and 3D analyses allow to discriminate non-human primate species depending on the most frequently consumed food items [6,9–11]. Dental microwear analyses also provide reliable information about individual and environmental characteristics. For example, microwear textures of roe deer (3D analysis) reflect dietary variation observed across both seasons and sexes [12]. Seasonal variation in diet is also detected on the dental microwear of wedge-capped capuchins (2D analysis) [13].

Microwear analyses of different tooth surfaces may provide different but complementary information about the chewing process and the food items ingested. The occlusal surface of molar teeth faces the opposite jaw and undergoes both abrasion and attrition during the chewing process, resulting from food-tooth and tooth-tooth contacts, respectively [4] and producing both scratches and pits on the enamel [14]. This surface may be subject to fast microwear turnover rates if animals rely on abrasive or chewy food items (e.g., in howlers and vervet monkeys [15,16]). In these cases, interpretations about the feeding ecology of the studied species may be influenced by the “Last Supper Effect” [17]. By contrast, the non-occlusal, buccal surface of molar teeth, especially its lower part, appears to be relevant to reconstruct animals’ diet because it should only interact with the food items consumed [18] leading to local tissue deformation or removal (abrasion), mainly in the form of scratches [19], as a result of the rolling of particles pushed by the cheek against enamel [20]. Buccal microwear patterns allow, for example, to discriminate primate species according to their broad type of diet (e.g., [21–23]). Experimental analyses have further shown a certain stability through time of these buccal microwear patterns [24,25]), at least in the absence of significant dietary shifts, suggesting that they are probably less subject to a “Last Supper Effect” than are occlusal microwear patterns [25].

In this study, we used a 2D dental microwear analysis to investigate the environmental and individual characteristics that influence *in vivo* buccal microwear patterns in a natural population of mandrills (*Mandrillus sphinx*) from Southern Gabon. Mandrills are generalist feeders relying mostly on fruits but also on various plant parts, as well as on invertebrates and vertebrates [26,27]. In the studied individuals, behavioral observations and a scale-sensitive fractal analysis of the 3D texture of occlusal dental microwear both indicate that diet largely varies seasonally, as well as between sexes and across ages [27]. In particular, mandrills consume tougher (e.g., plant leaves, roots, stems) and more underground food items (mainly plant roots) during the long dry season and more soft food items (mainly fruits) and monocotyledonous plants during the long rainy season [11]. Furthermore, males and older animals consume more hard food items than females and younger individuals while the later consume more monocotyledonous plants than older conspecifics [11]. Finally, tooth macrowear, characterized by topographical changes of the teeth (e.g., basin enlargements or modification of crest height and facet slopes [28]) and estimated by the percentage of dentine exposure (PDE), is strongly correlated with age in this primate population ([29]; and see in other species: [30,31]). In particular, mandrills’ molars appear to wear more rapidly (i.e., higher PDE for age) compared to savanna baboons [29]. In addition, older mandrills consume larger amounts of hard food items than younger individuals [11]. We anticipate that an important tooth macrowear in these old mandrills could facilitate the crushing of hard food items such as seeds and nuts because of enlarged basins on the molars [32]. We also hypothesize that buccal microwear patterns reflect mandrills’ feeding ecology. Consequently, we first expect these microwear patterns to be affected by the season of sampling as well as the individual’s sex and age because all these factors have concurrent effects on mandrills’ feeding strategies [11,27]. If verified, we also expect PDE to correlate with buccal microwear patterns because tooth macrowear may impose mechanical constraints during the chewing process, influencing individuals’ feeding strategies and, in turn, microwear.

## Materials and methods

### Studied population and behavioral analyses

This study was conducted on a free-ranging population of ~130 mandrills living in the Lékédi Park and surrounding areas (866 ha), in southern Gabon. This population originates from two groups of mandrills released in 2002 and 2006 (see [33] and [34] for details) and comprises both captive and wild born individuals, the latter representing more than 85% of the studied animals at the time of the study. Mandrills’ diet was improved with bananas and home-made cakes several times a week following the two release events. Provisioning decreased progressively throughout the years to completely cease in April 2012.

The studied mandrills live in a mosaic landscape mainly composed of closed equatorial forests but also humid open savannas and grasslands [33]. Soils of the Lékédi Park are characterized by a high concentration of quartz and the presence of kaolinite, gibbsite and undetermined clay [29]. Quartz from these sediments is proposed to be an aggravating factor of tooth macrowear in the studied mandrills by contrast with savanna baboons that feed on a less quartz-concentrated soil and experience more moderate tooth macrowear [29]. Gabon is characterized by four seasons: a long rainy season (Feb–May), a long dry season (Jun–Sep), a short rainy season (Oct–Nov) and a short dry season (Dec–Jan) (Abernethy et al 2002).

Feeding behavior data were collected on a daily basis between May 2013 and Oct 2014 using 5-min focal sampling of 57 individually recognized animals [27]. We kept individuals observed more than an hour per season to avoid non-representative data. We classified the 449 different consumed plant items into different categories according to their mechanical properties (via *in vivo* observations) and their abrasion potential–whether they could be highly concentrated in abrasive particles originated from plant tissues (phytoliths) (see [11]). These feeding data were paralleled with PDE values obtained on a subset of animals (see below).

### Dental molding and replicas production

Four trapping events occurred between April 2012 and July 2014 (during both long rainy and long dry seasons). During these captures, we obtained 149 *in vivo* dental molds on 88 anaesthetized, known individuals of all ages (ranging from 0.6 to 20.1 yrs) and both sexes. The trapped individuals were anaesthetized with a mix of ketamine and xylazine (Imalgène 1000 ® and Rompun®; see for details [34]) and awakened after 30–40 min using atipamezole (Antisedan ND, 0.5 mg/ml). We applied a silicone dental molding material (polyvinylsiloxane, PresidentJet regular body, Coltène® Corp; [35]) on post-canine teeth after a thorough cleaning (teeth were rinsed, brushed and dried). We latter produced high-resolution replicas from these silicon-based molds using a transparent epoxy resin (Araldite® 2020, Hunstman®), following validated protocols [36,37].

### Buccal microwear analysis

We studied a subset of 73 suitable replicas of mandrills’ molars with preserved buccal tooth surfaces and without imperfections caused by molding or cast processing, collected on 51 individuals (22 males aged 1.7–14.8 and 29 females aged 2.3–19.5; see S1 Table). We analyzed buccal microwear patterns of the first (M_1_) and second (M_2_) left lower molars. A ~15-nm layer of gold-palladium was laid on the epoxy replicas that were then examined under a scanning electron microscope (SEM) Hitachi S3000N (SS.TT. Investigación, Univ. Alicante) at a magnification varying between 100× and 120× depending on tooth dimensions and cleanliness [38]. The cement-enamel junction of each tooth was placed parallel to the SEM stub. We recorded SEM micrographs (1280×960 pixels, BMP file format) of buccal tooth surfaces, at the middle to upper thirds because lower parts were often covered with a patina layer [39]. SEM micrographs were obtained either on the mesial part or on the distal part of the buccal tooth surfaces. From the original images, we cropped square areas of 0.45 mm^2^ (671×671 μm) using Adobe Photoshop^TM^ 6.0, adjusted as a function of the initial magnification. As such, buccal microwear patterns were comparable across pictures. We applied a high-pass filter (50 pixels) and an automatic adjustment of color and tonal intensity levels [35,40].

Buccal microwear patterns were defined according to six variables calculated from the measurements of all non-ambiguous scratches retrieved from the SEM micrographs (clearly identifiable, longer than 15 μm, and at least four times longer than wider [39]) using a semi-automatic image analysis software (Sigma Scan ProV, SPSS^TM^). All the SEM micrographs were analyzed at least three times to improve the reliability of the measurements. For each micrograph, we obtained the total number of scratches, the average scratch length (in μm) and the buccal scratch densities by orientation (in degrees from 0 to 180), with the latter parameter decomposed into four microwear variables: we considered the number of scratches distributed within four distinct sub-areas of the studied tooth surface defined according to their orientation (from 0 to 180°) with respect to the cement-enamel junction of the tooth (S2 Table). These four sub-areas included scratches oriented in four 45° angle portions of the images, namely the horizontal, the disto-mesial (oriented from mesio-cervical to disto-occlusal part of the tooth), the vertical and the mesio-distal (from mesio-occlusal to disto-cervical) sub-areas (see for details: [19]). We multiplied by 100 the number of scratches following these different orientations and divided the result by the total number of scratches in order to obtain percentages of scratches of different orientations per sample.

In addition, we kept five microwear variables for a comparison of the buccal microwear patterns recorded in the studied mandrills with those recorded in other primates (from [22]). For these inter-population comparisons, we used a standardized value for the total number of scratches in order to limit potential biases due to differences in dimensions across the analyzed squared areas (covering, in our study, 0.45 mm^2^ of the buccal tooth surface instead of the usual 0.56 mm^2^ [22,25,35,36]). We analyzed the scratch density, calculated as the total number of scratches divided by the squared area dimensions of the different datasets, as well as the percentage of horizontal, oblique (disto-mesial or mesio-distal) and vertical scratches obtained by dividing the number of scratches in every orientation by the total number of scratches.

**Fig 1:**
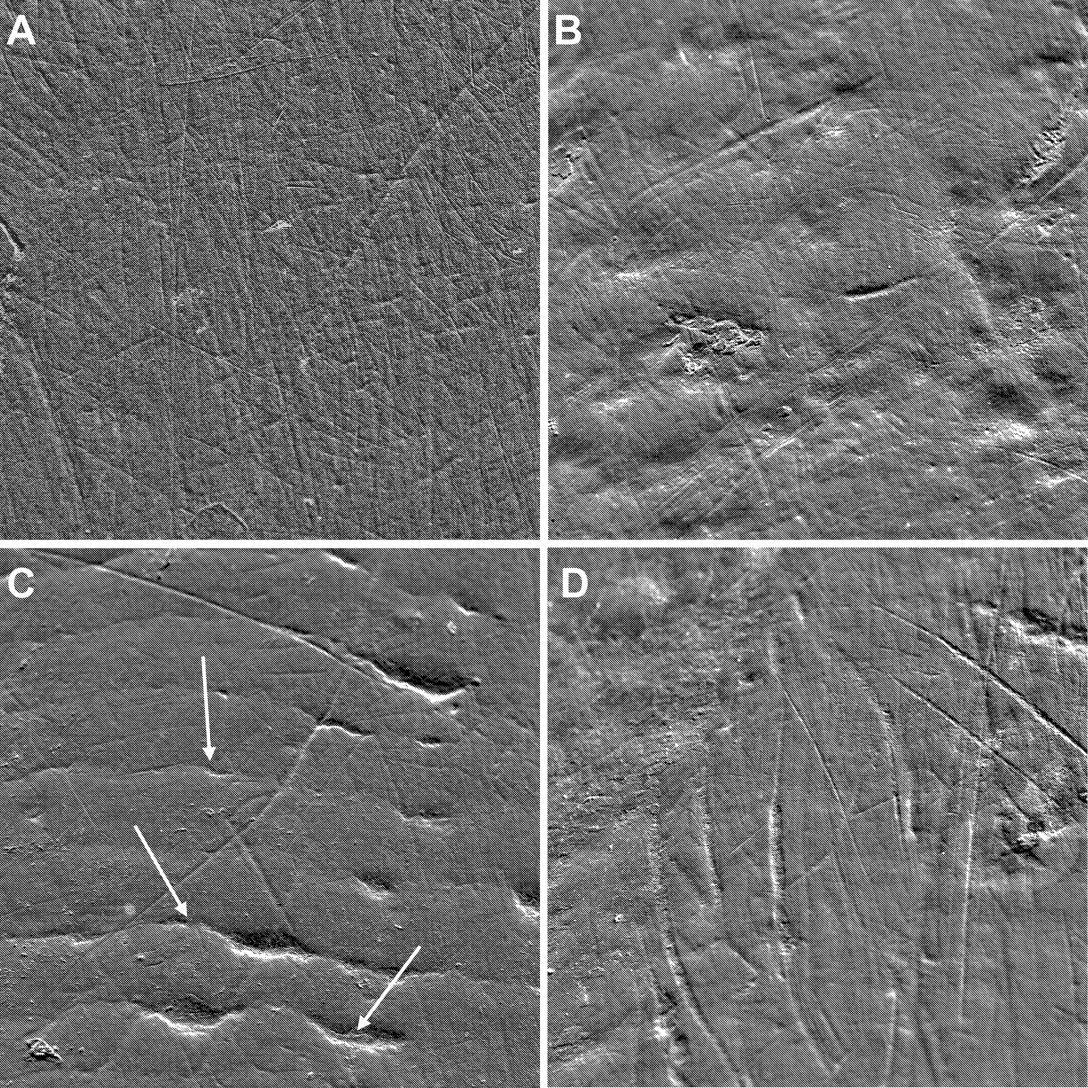
Buccal tooth surfaces (0.45 mm^2^) of mandrills from the studied population. The different SEM micrographs show different microwear patterns with (A) no evident artifacts; (B) some artifacts: patina layers and patches of erosion characterized by groups of pinholes; (C) visible perikymata paralleling the cement-enamel junction (indicated by the white arrows); and (D) with large scratches and fuzzy areas due to the curvature of the tooth surface.

Finally, we initially estimated the quality of each image used for all analyses using eight subjective variables (e.g., small artifacts, patches of erosion or visible perikymata; Fig 1). Detailed data and results are proposed as supplementary information (S3 Table). While the quality of these images slightly impacted buccal microwear patterns, it did not change the results we found.

### Measurement of the percent of dentine exposure (PDE)

Two-dimensional digital images (2592 × 1944 pixels) of the occlusal tooth surface of mandrills’ M_1_ were taken from the silicon based dental molds using a LEICA^®^ MZ 16 stereomicroscope. PDE was calculated as the area of exposed dentine divided by the area of the complete occlusal surface, using ImageJ. Complete occlusal surface comprised the whole visible crown, including molar rims that join the occlusal tooth surface to the gum. This measurement method allowed taking into account that the crown’s height as well as the cross section of the tooth may vary depending on tooth macrowear rate. Dental molds obtained in 2012 were used in a previous study [29] but re-analyzed in this study because our method slightly differed from the one used by Galbany and colleagues [29] to calculate PDE. Indeed, authors formerly analyzed photographs of replicas obtained from putty molds instead of silicone-based molds as we did. PDE measurements are provided in S4 Table.

### Statistical analyses

#### Inter-individual variation and microwear variable contribution

We performed a Principal Component Analysis (PCA, FactoMineR v. 1.32 [41], R v. 3.2.3) to explore inter-individual variation in microwear patterns of the 73 mandrills’ buccal tooth surfaces and to assess the relative weight of each of the six studied microwear variables on the overall variance.

#### Seasonal and individual effects on microwear variables

Using General Linear Mixed Models (LMM; nlme package v. 3.1–127 [42], R), we investigated the influence of the season of sampling (long dry season *vs*. long rainy season; class variable), individual’s age (continuous variable) and sex (female *vs*. male; class variable), as well as the sampled tooth (M_1_ or M_2_; class variable) and the tooth part (mesial or distal; class variable) on each of the six microwear variables (total number of scratches, average scratch length and scratch density for the four different orientations). We transformed some of these variables to fit to Gaussian distributions (Shapiro-Wilk tests; stats package, R) using either an inverse transformation (average scratch length) or a square root transformation (percentages of mesio-distal and vertical scratches). Individual’s age was determined using either exact birth dates, known for 15 captive-born individuals, or estimated birth dates for 36 wild-born individuals based on general body conditions and patterns of tooth eruption, with a possible estimated error of less than a year [29]. We used the individual’s identity as a random factor because most individuals were sampled more than once (1.45 on average, ranging from 1 to 4 times). Here and below, we always kept the full models as final models.

#### PDE and microwear variables

In these same six LMMs (same settings), we first replaced individual’s age by individual’s PDE because both variables were highly correlated (R^2^ = 0.88). In these models, we used a restricted dataset of 60 buccal tooth surfaces with well-preserved crowns. Second, we tested the effect of the residuals of PDE values not explained by individual’s age in the above six LMMs (along with individual’s age). These residuals were obtained using a lowess analysis (locally weighted scatterplot smoothing regression; stats package, R) performed between PDE and age.

#### PDE and diet variables

Data on feeding behavior was recorded between May 2013 and Oct 2014 while dental molding occurred in Apr 2012, Sep 2012, Apr 2013, and Jul 2014. We therefore obtained a combination of both behavioral data and dental molds (PDE measurements) for 30 individuals that provided 41 molds in April 2013 (N = 15) and July 2014 (N = 26). Because behavioral observations took place between these two trapping events, we were able to analyze both the impact of PDE on diet (April molds) and the impact of diet on PDE (July molds) using Spearman correlation tests. For these analyses, we considered the residuals of the PDE (not explained by age) obtained from the lowess analyses as well as each of the following diet variables: the proportion of hard (e.g., seeds, fruits with hard exocarps), soft (e.g., fruits, flowers), and tough (e.g., leaves, stems) food items as estimates of food mechanical properties, as well as the proportion of monocotyledonous plants consumed as an estimate of food abrasiveness because of their high phytolith content in comparison with dicotyledonous plants (see for details: [43]). These four diet variables were retrieved for all 30 animals by dividing the total number of consumption (occurrences) of each food category by the total time of focal observation [11,27]. We applied Holm-Bonferroni corrections to take into account possible biases due to multiple testing.

#### Inter-species comparisons

We compared buccal microwear patterns of the studied mandrills with those obtained on other primates (S5 Table), using a Principal Component Analysis based on the five selected microwear variables. The PCA allowed assessing the respective weight of each of these microwear variables on variation of buccal microwear patterns across the studied primate species. Moreover, this analysis allowed identifying the microwear variable(s) that better depict the diversity of ecological niches among these primates.

#### Ethics

Protocols used for our research have been validated by the “Centre National de la Recherche Scientifique et Technologique” (CENAREST, Gabon; authorization numbers: AR0001/14 and AR0018/15) and we obtained CITES permits to export biological material (permit numbers: 023/15, 024/15, 025/15). The research adhered to the legal requirements of Gabon for the ethical treatment of non-human primates and was further approved by the local ethic committee (#0020/2013/SG/CNE).

## Results

### Inter-individual variation and microwear variables’ contribution

The PCA used to evaluate the relative impact of the six studied microwear variables on the overall variance of buccal microwear patterns reveals that scratch densities by orientations better explain inter-individual variations. Indeed, the first principal component, accounting for 44.21% of the total variance is characterized by a positive load of the percentage of horizontal scratches (79%) and a negative load of the percentage of vertical scratches (−94%). The total number of scratches also shows a positive load (66%) and the other buccal microwear variables weigh less than 60%. The second principal component, that explains 24.36% of the total variance, mainly corresponds to oblique scratches where the percentage of mesio-distal scratches has a positive load (69%) and the percentage of disto-mesial scratches has a negative load (−85%). The third principal component, accounting for 13.19% of the total variance, is essentially characterized by a positive load of the average scratch length (46%) and a negative load of the total number of scratches (−53%).

### Effects of seasonality, individual characteristics and PDE on buccal microwear patterns

**Fig 2:**
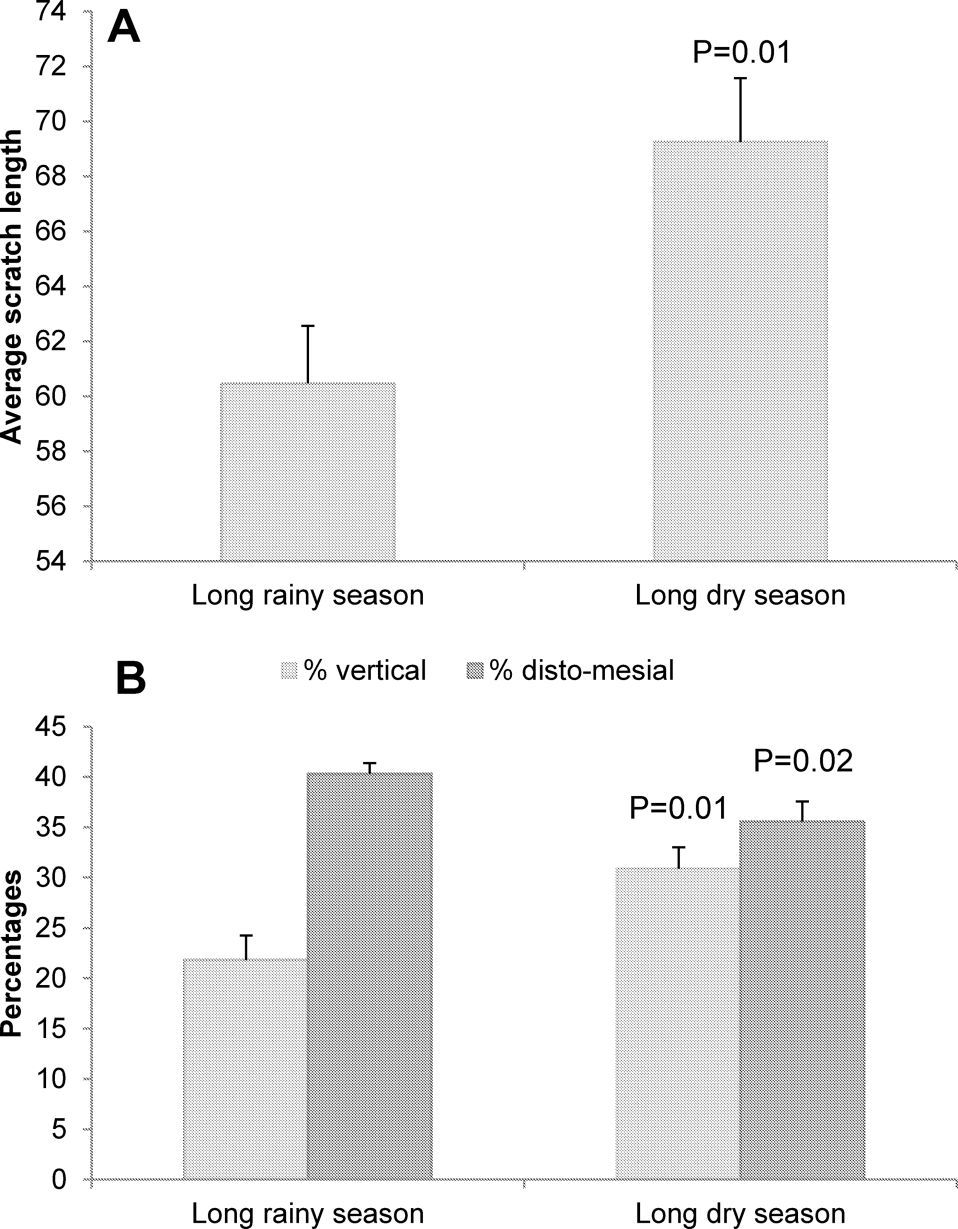
**Effects of the season of sampling on three buccal microwear variables: (A) the average scratch length; and (B) the percentages of vertical scratches and disto-mesial scratches.** Error bars indicate the standard error of the mean.

We show that buccal microwear patterns vary with seasonality, individual characteristics and with the tooth part but not with the tooth analyzed (LMM; Tables 1 and 2). First, the buccal tooth surface presents more scratches on the mesial part of the tooth compared to the distal part, although our data set was unbalanced (we compared 63 mesial parts to 10 distal parts). Second, the buccal tooth surface shows, on average, longer and more vertical scratches but fewer disto-mesial scratches during the long dry season than during the long rainy season (Fig 2; Table 1). Third, females present more disto-mesial scratches compared to males (Table 1). Finally, both age and PDE, but not the residuals of PDE (not explained by individual’s age), significantly or marginally positively correlate with the total number of scratches and the percentage of horizontal and mesio-distal scratches but negatively correlate with the percentage of vertical scratches (Table 3).

**Table 1:** Description of the six buccal microwear variables, by their means and standard deviations (±SD) calculated from samples collected during the full study period as well as during the two studied seasons, and also obtained separately from males and females.

	Overall N = 73	Long dry season N = 46	Long rainy season N = 27	Females N = 44	Males N = 29
**Total number of scratches**	253.93 (±100.3)	245.37 (±100.5)	268.5 (±100.1)	265.3 (±98.3)	236.66 (±102.6)
**Average scratch length**	66.01 (±14.6)	**69.26 (±15.7)**	**60.5 (±10.9)**	64.9 (±12.3)	67.77 (±17.7)
**% horizontal scratches**	20.55 (±7.9)	19.91 (±8.45)	21.6 (±6.8)	21.1 (±7.0)	19.73 (±9.1)
**% disto-mesial scratches**	37.33 (±8.6)	**35.58 (±7.1)**	**40.3 (±10.2)**	**39.5 (±7.3)**	**34.00 (±9.5)**
**% vertical scratches**	27.55 (±15.1)	**30.91 (±16.3)**	**21.8 (±10.9)**	25.8 (±14.3)	30.19 (±16.15)
**% mesio-distal scratches**	14.45 (±9.6)	13.42 (±9.4)	16.2 (±9.7)	13.6 (±8.8)	15.78 (±10.7)

Significant differences (P<0.05) observed between the long dry and the long rainy seasons (see Table 2) and between males and females are shown in bold.

**Table 2:** Effects of seasonality, individual characteristics, and tooth properties on buccal microwear patterns.

Microwear variables	Explanatory variables	F	P-value
**Total number of scratches**	Season	2.23	0.15
	Sex	0.84	0.36
	**Age**	**10.92**	**<0.01**
	Tooth	1.01	0.33
	**Tooth part**	**5.54**	**0.03**
**Average scratch length**	**Season**	**8.48**	**0.01**
	Sex	0.05	0.82
	Age	2.17	0.16
	Tooth	<0.001	1.00
	Tooth part	1.77	0.20
**% mesio-distal scratches**	Season	2.51	0.13
	Sex	1.13	0.29
	Age	3.23	0.09
	Tooth	0.13	0.72
	Tooth part	1.12	0.30
**% vertical scratches**	**Season**	**9.95**	**0.01**
	Sex	0.77	0.38
	**Age**	**6.20**	**0.02**
	Tooth	<0.01	1.00
	Tooth part	0.73	0.40
**% horizontal scratches**	Season	1.91	0.18
	Sex	0.07	0.80
	**Age**	**6.56**	**0.02**
	Tooth	<0.001	0.99
	Tooth part	1.49	0.24
**% disto-mesial scratches**	**Season**	**6.72**	**0.02**
	**Sex**	**7.31**	**0.01**
	Age	0.02	0.90
	Tooth	0.19	0.67
	Tooth part	0.48	0.50

Results from the LMM are provided (F and P-values) and significant effects (P<0.05) are shown in bold.

**Table 3:** Effects of PDE on buccal microwear patterns.

Microwear variables	PDE variables	F	P-value
**Total number of scratches**	**PDE**	**7.29**	**0.02**
	Residuals of PDE	0.01	0.76
**Average scratch length**	PDE	0.09	0.77
	Residuals of PDE	0.03	0.87
**% mesio-distal scratches**	**PDE**	**4.59**	**0.05**
	Residuals of PDE	0.34	0.57
**% vertical scratches**	**PDE**	**8.07**	**0.01**
	Residuals of PDE	0.01	0.94
**% horizontal scratches**	**PDE**	**9.10**	**0.01**
	Residuals of PDE	1.50	0.24
**% disto-mesial scratches**	PDE	0.01	0.91
	Residuals of PDE	0.68	0.43

### PDE and diet variables

We show that PDE, independently of individual’s age, is not correlated with the proportion of consumption of hard, soft and tough food items either before or after dental molding (Table 4). However, PDE and the proportion of monocotyledonous plants consumed before dental molding are significantly positively correlated, while this relationship disappears when considering the proportion of monocotyledons consumed after molding (Table 4).

**Table 4:** Relationships between PDE and diet variables.

Diet variable	Residuals of PDE before behavioral observations		Residuals of PDE after behavioral observations	
	R^2^	P-value	R^2^	P-value
**% Hard food items**	0.31	0.02	0.05	0.33
**% Soft food items**	0.21	0.09	0.10	0.15
**% Tough food items**	0.01	0.69	0.01	0.70
**% Monocotyledons**	0.38	0.04	**0.32**	**<0.01**

Results of Spearman correlation tests are displayed (R^2^ and P-values). Significant correlations after sequential Holm-Bonferroni corrections are highlighted in bold.

### Inter-species comparisons

**Fig 3:**
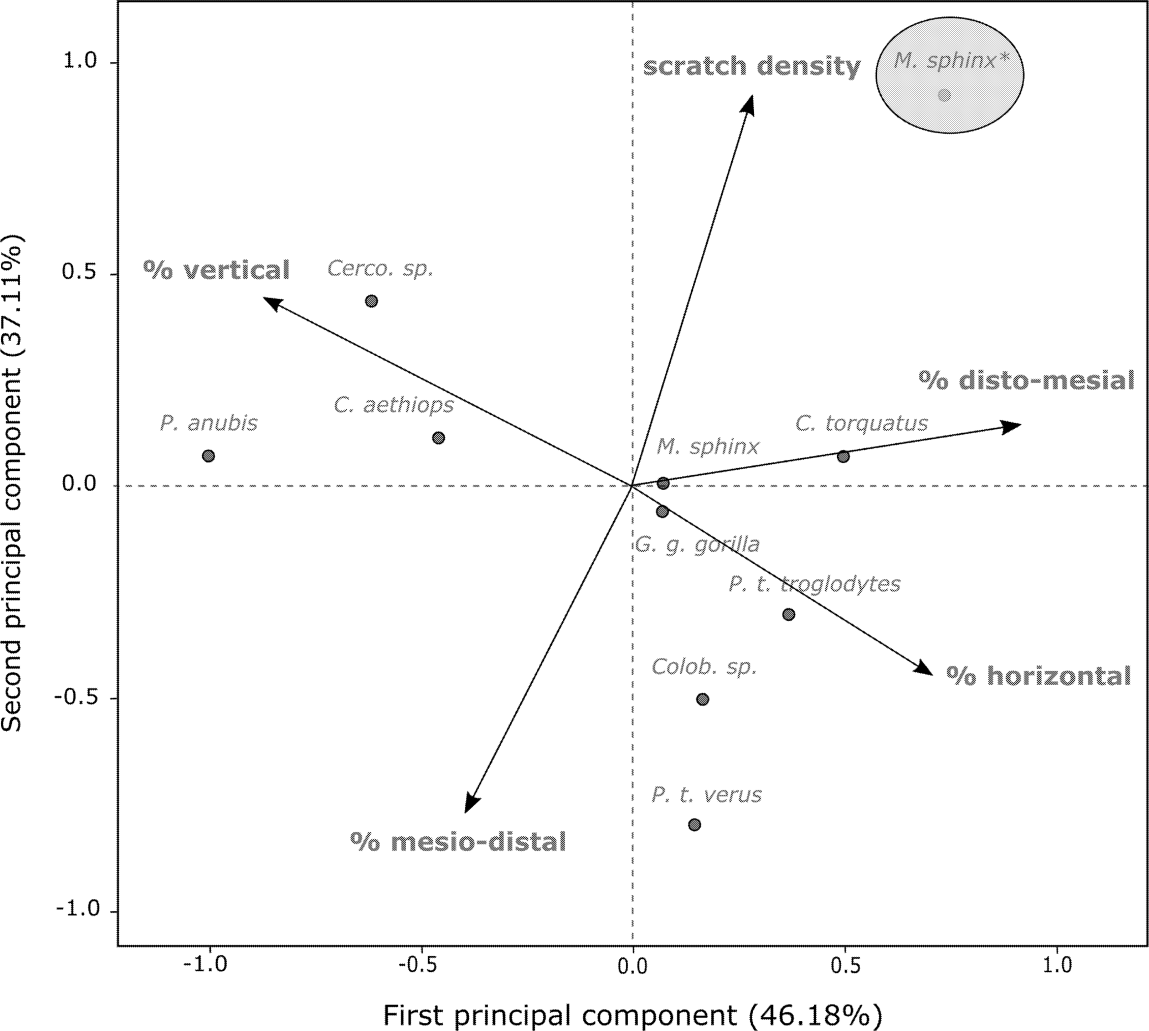
First (PC1) and second (PC2) principal components of the PCA showing differences in buccal microwear patterns across several primate species. The studied mandrill population is highlighted (circle). The labeled arrows show the unrotated loadings of microwear parameters onto PC1 and PC2. Comparative microwear data are obtained from Estebaranz and colleagues [22].

The comparison between the buccal microwear patterns of different primate species reveals that the study population of mandrills highly deviates from other primate species along the second principal component (PC2; 37.11% of the total variance), while in their range regarding the first principal component (PC1; accounting for 46.18% of the total variance; Fig 3). PC1 is essentially characterized by positive loads of the percentages of disto-mesial and horizontal scratches (91% and 70% resp.) and a negative load of the percentage of vertical scratches (−87%), whereas PC2 is well-defined by a positive load of the scratch density (93%) and a negative load of the percentage of mesio-distal scratches (−76%; See S5 Table).

## Discussion

In contrast to microwear patterns obtained from the occlusal tooth surface, the relevance of buccal microwear patterns to study the feeding ecology of animal species has been questioned [17]. Yet, several studies demonstrated that buccal microwear analyses can e.g., discriminate primates species according to their consumption of hard brittle or tough food items [23] or indicate the amounts of abrasive foods consumed across different human societies [35]. In this study, we show that buccal scratch densities by orientation contribute the most to inter-individual variation in mandrills’ buccal microwear patterns. We further find correlation relationships between these patterns and the season of sampling as well as individual’s age and sex. In addition, PDE and age tend to produce similar patterns on the buccal tooth surfaces while PDE, independently of individual’s age, seems to reflect short-term variation of individual’s diet. Finally, our inter-species comparison shows that mandrills group together with generalist feeders with a frugivorous tendency.

### Seasonality and individual characteristics

Almost all studied microwear variables vary with the season of sampling and/or individual characteristics: the long dry season is characterized by longer and more vertical scratches but less disto-mesial scratches than during the long rainy season and males also present less disto-mesial scratches than females. Individual’s age is probably the variable impacting the most buccal scratch patterns with four different microwear variables involved: older animals show more scratches overall, especially more mesio-distal and more horizontal scratches, although less vertical scratches, than younger mandrills.

Regarding seasonality, our results contrast with prior studies on medieval agriculturalist human populations, where seasonal variations are not detected on buccal tooth surfaces [16]. The turn-over rate of buccal microwear has been shown to little vary (±2.5 scratches/week) in modern humans feeding on natural, non-induced food resources [19]. The seasonal variation detected in mandrills suggests that turn-over rates in this population may be larger than those described in these human populations, possibly in relation to different feeding ecologies that may also depend on tooth shape.

In other study systems, the total number of scratches is often used to relate variation in buccal microwear patterns to diet because this variable has been proposed to reflect abrasiveness of the food items consumed [19,22,29]. Surprisingly, in our study, the total number of scratches is only a poor predictor of seasonality and individual characteristics (with the exception of individual’s age). This variable should constitute, as such, a weak proxy for dietary variation because, in the study population, diet largely varies both seasonally and individually [11]. By contrast, scratch densities by orientation (vertical, horizontal, mesio-distal and disto-mesial) appear to be better related to these seasonal and individual variations. In cercopithecoid and hominoid species, for example, these variables have been shown to discriminate well between dietary groups [35,44]. While we do not have usable overlapping data between buccal microwear patterns and feeding behavioral data on the studied mandrills, we suspect possible relationships between food physical properties (mechanical properties, abrasiveness) and buccal scratch densities by orientation. For example, mandrills’ buccal tooth surfaces present longer scratches during the long dry season compared to the long rainy season. During dry weather, mandrills are also known to consume more tough and underground food items (covered of abrasive grit; [45]). These food items may require longer chewing cycles, involving sliding movements between the upper and the lower jaws and resulting in longer scratches, if longer scratches correspond to prolonged contacts between food particles and the buccal tooth surface (as per: [36]). Differences in jaw kinematics may also explain the numerous correlations found between individual’s age and microwear variables. Dietary differences have also been highlighted between old and young mandrills (e.g., an increase of hard food items consumption with ageing; [11]). While diet composition is probably not the sole factor involved in buccal scratch patterns observed in mandrills, our results suggest that jaw kinematics may differ depending on food mechanical properties ([46–49]; but see: [50]), leading to different buccal scratch patterns. Indeed, jaw movements have been suggested to impact scratch densities by orientation in cercopithecids and humans [36,51]. Detailed analyses on jaw kinematics analyses are now required to further the discussion.

### PDE, diet and buccal microwear patterns

In this study, we show that old mandrills have both high PDE and high (total) number of scratches and that these two dental variables appear, in turn, positively correlated. These findings suggest that ageing leads to both tooth macrowear and the accumulation of scratches on the buccal tooth surface (see discussion in: [52]). Alternatively, the mechanical constraints possibly imposed by PDE during a lifetime may lead old individuals to perform more chewing cycles than younger animals to consume a given food item, a strategy observed in koalas [53]. In the studied mandrills, PDE and age show similar impacts on buccal microwear patterns. Inter-individual variation in PDE regardless of mandrills’ age seems, however, not important enough to drive changes in buccal microwear patterns, through differentiated individual feeding strategies or jaw kinematics. In line with this, we find that PDE does not impact the food items consumed by the mandrills after dental molding, suggesting that it does not spearhead variations in individual feeding strategies. This result is probably not surprising because only critical tooth macrowear is expected to involve significant changes in an individual’s diet. This is consistent with the findings that the first molars of savanna baboons do not present more advanced stages of wear when individuals spend more time feeding on fruits, leaves or seeds [30]. PDE, in these baboons, correlates, however, with the percent of time spent feeding on grass corms that are highly covered by underground (abrasive) grit. While the studied mandrills consume underground food items in limited quantities (but see discussion about quartz contained in the soils of the Lékédi park in: [29]), they feed on monocotyledonous plants that are thought to contain high concentration of abrasive phytoliths [41]. Interestingly, we show that the proportion of monocotyledonous plants consumed before dental molding correlates with high values of PDE possibly because these plants amplify enamel abrasion [54].

### Inter-species comparisons

Results from the inter-specific comparison show that the studied population of mandrills presents similar average values on the PC1 axis, mainly characterized by the percentages of disto-mesial, vertical and horizontal scratches, than other primate species, such as *Colobus sp*., *Pan troglodytes troglodytes*, *P*. *t*. *verus* and *Gorilla gorilla*. Almost all these species are generalist feeders with a tendency towards frugivory [22]. Additionally, on this axis, the studied mandrills appear close to a small sample of mandrill specimens obtained from Museum collections but they are even closer to a population of *Cercocebus torquatus*. Interestingly, *Cercocebus* and *Mandrillus* genera are phylogenetically very close to each other [55] and share similar geographical range and feeding habits [56,57]. Similarities between both buccal microwear patterns and geographical ranges are also reported in two sympatric great apes (*P*. *troglodytes troglodytes* and *G*. *gorilla gorilla* [40]). Finally, scratch density highly discriminates the studied mandrills from other primates along the PC2 axis, possibly highlighting biases resulting from comparisons across studies that probably differed in their methodologies or data collection. Altogether the results we obtained from the two principal components analyses performed at the intra- and at the inter-species levels both indicate that buccal scratch densities by orientation account for the largest proportion of the variance observed within and between different primate species and should be considered in future comparative studies because of their high discriminatory potential.

## Concluding remarks

Although dental microwear have been studied for decades in mammals [e.g., 1, 2, 18, 19, 58, 59], processes involved in their formation remain poorly understood and are sometimes contradictory. In particular, the roles of food mechanical properties, abrasive particles arising from aerial dust and/or grit and jaw kinematics on the formation of dental microwear patterns are still largely debated [54,60–68]. While our results possibly indicate that buccal microwear patterns are informative regarding general changes in mandrills’ diet, we need now to confirm our assumptions using e.g., direct analyses of the relationships between an individual’s feeding behavior and its microwear patterns (with data collected at the same time), or mechanical analyses of the food items consumed and individual’s jaw kinematics.

## Supporting information

S1 Table Information about the buccal tooth surfaces analyzed in this study. Details about individual’s sex, age and date of capture are provided. This table also indicates if every dental mold was used for PDE analyses. All the dental molds and replicas are stored in the Institut des Sciences de l’Evolution de Montpellier, at the University of Montpellier, France.
(DOC)

S2 Table Buccal microwear data for the 73 SEM micrographs analyzed in this study. (DOCX)

S3 Table Effects of image quality on buccal microwear variables. Eight qualitative scores were attributed to each analyzed SEM micrograph (degree of fuzziness or erosion, patina, presence of highly curved scratches, holes, artifacts or pits, presence of visible perikymata). A total of 48 LMM were run to study their individual effects on each of the six studied buccal microwear variables. These models included the main variables described in the main text (seasonality, individuals’ age and sex, tooth identity and tooth part). For clarity sake, we present only significant results.
The fuzziness score ranges from 1 (image not fuzzy) to 3 (between 25 and 50% of the image is fuzzy); the erosion score ranges from 1 (image not eroded) to 3 (three and more patches of erosion or one large patch of erosion covering ~10% of the image); the presence of highly curved scratches (measured in different segments) ranges from 1 (no scratch is highly curved) to 3 (at least five scratches are highly curved).
(DOCX)

S4 Table Detailed information about individuals for which both feeding behavior and PDE data are available. These individuals were used to analyze the relationships between PDE and diet (Spearman correlation tests). Date of dental molding, individual’s age, PDE, residuals of PDE (independent of age) and percentages (noted “%”) of food items consumed according to their physical properties are provided in this table. The physical properties tested in this study are encoded as hard, soft and tough food items (mechanical properties) and monocotyledonous plants, where this plant clade indicates, *a priori*, a high concentration in phytoliths (food abrasiveness).
(DOCX)

S5 Table Comparison of buccal scratch patterns between the studied mandrills and other primates, considering average values for five microwear variables based on Estebaranz and colleagues (2012). For the sake of clarity, the mandrill population we studied is highlighted (*), by contrast with the other mandrill population, which corresponds to four samples collected in a museum (Estebaranz et al 2012).
(DOCX)
