# Supplementary material for: Buccal dental-microwear and dietary ecology in a free-ranging population of mandrills (Mandrillus sphinx) from southern Gabon
Source: PLoS One. 2017 Oct 26;12(10):e0186870. doi: 10.1371/journal.pone.0186870 (PMC5658090; doi:10.1371/journal.pone.0186870)
Supplement: S1 Table — Details about individual’s sex, age and date of capture are provided. This table also indicates if every dental mold was used for PDE analyses. All the dental molds and replicas are stored in the Institut des Sciences de l’Evolution de Montpellier, at the University of Montpellier, France. (DOC) [file pone.0186870.s001.doc]

**S1 Supporting information**

**S1 Table. Information about the buccal tooth surfaces analyzed in this study.** Details about individual’s sex. age and date of capture are provided. This table also indicates if every dental mold was used for PDE analyses. All the dental molds and replicas are stored in the Institut des Sciences de l’Evolution de Montpellier. at the University of Montpellier. France.

| SEM micrograph ID | Individual’s identity | Sex | Age | Date of capture | Tooth analyzed | Part of the tooth | Analysis PDE |
| --- | --- | --- | --- | --- | --- | --- | --- |
| M002-apr2013-M1 | 2 | Male | 3.15 | 25/04/2013 | M1 | mesial | yes |
| M004-apr2013-M2 | 4 | Male | 5.66 | 30/04/2013 | M2 | mesial | yes |
| F007-sep2012-M2 | 7 | Female | 19.5 | 17/09/2012 | M2 | mesial | yes |
| M009-jul2014-M2 | 9 | Male | 5.85 | 09/07/2014 | M2 | mesial | yes |
| M011-mar2014-M1 | 11 | Male | 2.84 | 03/07/2014 | M1 | distal |  |
| M012-sep2012-M1 | 12 | Male | 8.05 | 18/09/2012 | M1 | mesial |  |
| M012-apr2013-M2 | 12 | Male | 8.48 | 24/04/2013 | M2 | mesial | yes |
| F013-apr2013-M1 | 13 | Female | 12.34 | 24/04/2013 | M1 | distal | yes |
| F013-jul2014-M2 | 13 | Female | 13.52 | 01/07/2014 | M2 | mesial | yes |
| F016-sep2012-M2- | 16 | Female | 8.5 | 17/09/2012 | M2 | distal | yes |
| F017-apr2012-M1 | 17 | Male | 4.04 | 15/04/2012 | M1 | mesial |  |
| F018-sep2012-M1 | 18 | Female | 4.06 | 22/09/2012 | M1 | mesial | yes |
| F018-apr2013-M2 | 18 | Female | 4.65 | 27/04/2013 | M2 | mesial | yes |
| F018-jul2014-M1 | 18 | Female | 5.83 | 03/07/2014 | M1 | mesial | yes |
| F019-apr2012-M2 | 19 | Female | 14.08 | 11/04/2012 | M2 | mesial | yes |
| F020-apr2012-M2 | 20 | Female | 9.86 | 11/04/2012 | M2 | mesial | yes |
| F020-sep2012-M2 | 20 | Female | 10.31 | 21/09/2012 | M2 | mesial | yes |
| F021-jul2014-M2 | 21 | Female | 15.16 | 09/07/2014 | M2 | mesial | yes |
| F023-apr2013-M1 | 23 | Female | 10.24 | 24/04/2013 | M1 | mesial | yes |
| F023-jul2014-M1 | 23 | Female | 11.43 | 01/07/2014 | M1 | mesial | yes |
| M024-jul2014-M1 | 24 | Male | 2.59 | 04/07/2014 | M1 | mesial | yes |
| M027-apr2013-M1 | 27 | Male | 2.65 | 25/04/2013 | M1 | mesial |  |
| M027-jul2014-M1 | 27 | Male | 3.83 | 02/07/2014 | M1 | mesial |  |
| F029-apr2012-M2 | 29 | Female | 13.18 | 15/04/2012 | M2 | distal | yes |
| F029-sep2012-M1 | 29 | Female | 13.61 | 17/09/2012 | M1 | mesial | yes |
| F029-apr2013-M1 | 29 | Female | 14.21 | 27/04/2013 | M1 | mesial | yes |
| F029-jul2014-M2 | 29 | Female | 15.4 | 03/07/2014 | M2 | distal | yes |
| F030-apr2013-M2 | 30 | Female | 10.9 | 28/04/2013 | M2 | distal | yes |
| F031-apr2013-M2 | 31 | Female | 7.65 | 25/04/2013 | M2 | mesial | yes |
| F031-jul2014-M2 | 31 | Female | 8.85 | 09/07/2014 | M2 | mesial | yes |
| F034-apr2012-M1 | 34 | Female | 3.04 | 15/04/2012 | M1 | mesial | yes |
| F034-apr2013-M1 | 34 | Female | 4.06 | 24/04/2013 | M1 | mesial | yes |
| M036-sep2012-M2 | 36 | Male | 13.05 | 18/09/2012 | M2 | mesial | yes |
| M036-apr2013-M1 | 36 | Male | 13.65 | 24/04/2013 | M1 | mesial | yes |
| F038-sep2012-M2 | 38 | Female | 10.64 | 21/09/2012 | M2 | mesial | yes |
| F039-jul2014-M2 | 39 | Female | 6.84 | 05/07/2014 | M2 | mesial | yes |
| F040-sep2012-M1 | 40 | Female | 7.05 | 19/09/2012 | M1 | mesial | yes |
| F040-jul2014-M1 | 40 | Female | 8.85 | 09/07/2014 | M1 | mesial | yes |
| F041-jul2014-M2 | 41 | Female | 6.83 | 02/07/2014 | M2 | mesial | yes |
| F042-apr2013-M1 | 42 | Female | 3.66 | 29/04/2013 | M1 | mesial | yes |
| F042-jul2014-M2 | 42 | Female | 4.84 | 06/07/2014 | M2 | distal |  |
| F043-apr2013-M1 | 43 | Female | 3.39 | 24/04/2013 | M1 | mesial |  |
| F043-jul2014-M1 | 43 | Female | 4.58 | 01/07/2014 | M1 | mesial | yes |
| F045-jul2014-M2 | 45 | Female | 13.66 | 06/07/2014 | M2 | mesial | yes |
| F047-jul2014-M1 | 47 | Female | 12.52 | 10/07/2014 | M1 | mesial |  |
| F048-apr2013-M2 | 48 | Female | 12.5 | 25/04/2013 | M2 | distal | yes |
| F048-jul2014-M1 | 48 | Female | 13.68 | 01/07/2014 | M1 | mesial | yes |
| M050-may2013-M1 | 50 | Male | 1.67 | 02/05/2013 | M1 | mesial | yes |
| M051-apr2013-M1 | 51 | Male | 4.06 | 24/04/2013 | M1 | mesial | yes |
| M053-sep2012-M1 | 53 | Male | 10.8 | 18/09/2012 | M1 | mesial |  |
| M054-jul2014-M2 | 54 | Male | 14.83 | 02/07/2014 | M2 | mesial | yes |
| F056-sep2012-M2 | 56 | Female | 11.64 | 22/09/2012 | M2 | mesial | yes |
| F056-jul2014-M1 | 56 | Female | 13.43 | 08/07/2014 | M1 | mesial | yes |
| M057-apr2012-M1 | 57 | Male | 8.12 | 11/04/2012 | M1 | mesial | yes |
| M057-sep2012-M2 | 57 | Male | 8.57 | 20/09/2012 | M2 | mesial | yes |
| M058-apr2012-M1 | 58 | Male | 5.04 | 14/04/2012 | M1 | mesial | yes |
| M058-apr2013-M1 | 58 | Male | 6.08 | 30/04/2013 | M1 | mesial | yes |
| M058-jun2014-M2 | 58 | Male | 7.25 | 30/06/2014 | M2 | mesial | yes |
| F059-jul2014-M1 | 59 | Female | 2.25 | 02/07/2014 | M1 | mesial |  |
| M066-apr2013-M1 | 66 | Male | 4.08 | 29/04/2013 | M1 | mesial | yes |
| M066-jun2014-M2 | 66 | Male | 5.25 | 30/06/2014 | M2 | mesial |  |
| M069-jul2014-M1 | 69 | Male | 3.26 | 05/07/2014 | M1 | mesial | yes |
| F070-jul2014-M1 | 70 | Female | 2.59 | 04/07/2014 | M1 | mesial | yes |
| F071-jul2014-M2 | 71 | Female | 4.84 | 03/07/2014 | M2 | mesial | yes |
| M074-jun2014-M2 | 74 | Male | 13.25 | 30/06/2014 | M2 | mesial | yes |
| M075-jun2014-M1 | 75 | Male | 7.49 | 30/06/2014 | M1 | mesial | yes |
| F076-jul2014-M1 | 76 | Female | 4.25 | 01/07/2014 | M1 | mesial |  |
| F081-jul2014-M1 | 81 | Female | 6.84 | 06/07/2014 | M1 | mesial | yes |
| M086-jun2014-M2 | 86 | Male | 2.25 | 30/06/2014 | M2 | distal |  |
| F100-jul2014-M2 | 100 | Female | 2.25 | 03/07/2014 | M2 | distal | yes |
| M103-jul2014-M1 | 103 | Male | 2.51 | 05/07/2014 | M1 | mesial | yes |
| F106-jul2014-M1 | 106 | Female | 4.52 | 09/07/2014 | M1 | mesial | yes |
| M111-apr2013-M1 | 111 | Male | 1.91 | 28/04/2013 | M1 | mesial | yes |
