# Supplementary material for: Buccal dental-microwear and dietary ecology in a free-ranging population of mandrills (Mandrillus sphinx) from southern Gabon
Source: PLoS One. 2017 Oct 26;12(10):e0186870. doi: 10.1371/journal.pone.0186870 (PMC5658090; doi:10.1371/journal.pone.0186870)
Supplement: S2 Table — (DOCX) [file pone.0186870.s002.docx]

**S2 Supporting Information**

**S2 Table. Buccal microwear data for the 73 SEM micrographs analyzed in this study.**

| SEM micrograph ID | % mesio-distal | % vertical | % horizontal | % disto-mesial | Total number of scratches | Average scratch length | PDE |
| --- | --- | --- | --- | --- | --- | --- | --- |
| M002-apr2013-M1 | 13.61 | 40.82 | 23.13 | 22.45 | 147 | 71.85 | 2.9 |
| M004-apr2013-M2 | 4.72 | 40.25 | 17.3 | 37.74 | 318 | 56.04 | 6.37 |
| F007-sep2012-M2 | 4.21 | 36.32 | 17.89 | 41.58 | 380 | 49.41 | 66 |
| M009-jul2014-M2 | 36.73 | 13.27 | 19.39 | 30.61 | 294 | 64.65 | 13.19 |
| M011-mar2014-M1 | 7.69 | 44.76 | 10.49 | 37.06 | 143 | 51.26 | - |
| M012-sep2012-M1 | 4.17 | 47.92 | 9.72 | 38.19 | 144 | 59.27 | - |
| M012-apr2013-M2 | 11.58 | 32.11 | 21.05 | 35.26 | 190 | 47.01 | 16.81 |
| F013-apr2013-M1 | 2.47 | 30.86 | 12.35 | 54.32 | 81 | 54.37 | 70.64 |
| F013-jul2014-M2 | 14.97 | 7.22 | 34.49 | 43.32 | 374 | 61.17 | 68.43 |
| F016-sep2012-M2- | 0.66 | 69.54 | 1.99 | 27.81 | 151 | 62.85 | 43.01 |
| F017-apr2012-M1 | 11.98 | 26.5 | 15.21 | 46.31 | 434 | 59.84 | - |
| F018-sep2012-M1 | 3.91 | 42.35 | 17.08 | 36.65 | 281 | 57.24 | 6.96 |
| F018-apr2013-M2 | 11.51 | 36.69 | 16.55 | 35.25 | 139 | 67.82 | 5.48 |
| F018-jul2014-M1 | 10.47 | 37.7 | 15.71 | 36.13 | 191 | 58.38 | 12.88 |
| F019-apr2012-M2 | 6.86 | 15.8 | 22.04 | 55.3 | 481 | 52.05 | 78.6 |
| F020-apr2012-M2 | 16.26 | 20.86 | 22.09 | 40.8 | 326 | 62.73 | 24.06 |
| F020-sep2012-M2 | 5.53 | 20.85 | 23.4 | 50.21 | 235 | 59.09 | 34.3 |
| F021-jul2014-M2 | 34.21 | 14.66 | 18.8 | 32.33 | 266 | 70.56 | 90.18 |
| F023-apr2013-M1 | 9.44 | 15.56 | 23.33 | 51.67 | 180 | 50.86 | 47.63 |
| F023-jul2014-M1 | 6.88 | 26.25 | 24.38 | 42.5 | 160 | 64.95 | 46.4 |
| M024-jul2014-M1 | 1.35 | 56.31 | 11.26 | 31.08 | 222 | 86.25 | 0.78 |
| M027-apr2013-M1 | 30.99 | 19.72 | 26.06 | 23.24 | 142 | 61.56 | - |
| M027-jul2014-M1 | 12.26 | 41.15 | 19.27 | 18.75 | 192 | 97.59 | - |
| F029-apr2012-M2 | 12.98 | 21.37 | 23.66 | 41.98 | 262 | 55.49 | 58.74 |
| F029-sep2012-M1 | 17.75 | 25.84 | 20.45 | 35.96 | 445 | 66.17 | 70.09 |
| F029-apr2013-M1 | 21.05 | 19.3 | 23.1 | 36.55 | 342 | 89.59 | 79.55 |
| F029-jul2014-M2 | 14.39 | 30.94 | 13.67 | 41.01 | 139 | 66.39 | 77.49 |
| F030-apr2013-M2 | 16.93 | 9.06 | 28.35 | 45.67 | 254 | 58.02 | 54.11 |
| F031-apr2013-M2 | 36.2 | 9.82 | 23.31 | 30.67 | 163 | 72.63 | 33.41 |
| F031-jul2014-M2 | 2.62 | 49.06 | 16.1 | 32.21 | 267 | 73.63 | 43.96 |
| F034-apr2012-M1 | 19.82 | 27.75 | 11.89 | 40.53 | 227 | 54.22 | 0.42 |
| F034-apr2013-M1 | 14.29 | 4.86 | 32.29 | 48.57 | 350 | 44.88 | 2.47 |
| M036-sep2012-M2 | 15.31 | 22.49 | 33.01 | 29.19 | 209 | 64.3 | 45.87 |
| M036-apr2013-M1 | 39.72 | 10.75 | 32.48 | 17.06 | 428 | 63.17 | 69.51 |
| F038-sep2012-M2 | 9.29 | 28.42 | 24.04 | 38.25 | 183 | 60.9 | 53.39 |
| F039-jul2014-M2 | 25.96 | 15.85 | 21.86 | 36.34 | 366 | 62.58 | 37.24 |
| F040-sep2012-M1 | 30.68 | 13.15 | 28.29 | 27.89 | 251 | 75.97 | 19.11 |
| F040-jul2014-M1 | 13.51 | 18.43 | 21.38 | 46.68 | 407 | 68.78 | 28.17 |
| F041-jul2014-M2 | 4.03 | 52.42 | 15.32 | 28.23 | 124 | 71.33 | 14.96 |
| F042-apr2013-M1 | 7.69 | 17.52 | 24.36 | 50.43 | 234 | 61.29 | 2.82 |
| F042-jul2014-M2 | 12.36 | 25.09 | 24.36 | 38.18 | 275 | 55.35 | - |
| F043-apr2013-M1 | 16.27 | 24.21 | 14.29 | 45.24 | 252 | 73.4 | - |
| F043-jul2014-M1 | 3.38 | 36.23 | 19.32 | 41.06 | 207 | 75.48 | 6.47 |
| F045-jul2014-M2 | 19.3 | 14.04 | 25 | 41.67 | 228 | 51.11 | 78.62 |
| F047-jul2014-M1 | 10.84 | 36.14 | 26.2 | 26.81 | 332 | 62.78 | - |
| F048-apr2013-M2 | 31.25 | 10.23 | 24.72 | 33.81 | 352 | 56 | 56.6 |
| F048-jul2014-M1 | 21.78 | 13.2 | 22.11 | 42.9 | 303 | 58.8 | 66.08 |
| M050-may2013-M1 | 7.57 | 47.32 | 7.57 | 37.54 | 317 | 48.23 | 0.88 |
| M051-apr2013-M1 | 4.67 | 11.33 | 31.33 | 52.67 | 150 | 81.98 | 2.91 |
| M053-sep2012-M1 | 30.59 | 24.05 | 18.78 | 26.58 | 474 | 53.47 | - |
| M054-jul2014-M2 | 16.36 | 23.64 | 23.64 | 36.36 | 165 | 68.47 | 72.37 |
| F056-sep2012-M2 | 15.57 | 18.58 | 21.58 | 44.26 | 366 | 77.42 | 47.59 |
| F056-jul2014-M1 | 17.04 | 2.69 | 36.77 | 43.5 | 446 | 68 | 61.43 |
| M057-apr2012-M1 | 12.17 | 17.68 | 28.7 | 41.45 | 345 | 59.66 | 23.41 |
| M057-sep2012-M2 | 27.22 | 6.96 | 33.86 | 31.96 | 316 | 54.17 | 30.02 |
| M058-apr2012-M1 | 11.38 | 30 | 15.17 | 43.45 | 290 | 46.24 | 7.66 |
| M058-apr2013-M1 | 27.86 | 15.79 | 31.89 | 24.46 | 323 | 58.39 | 8.73 |
| M058-jun2014-M2 | 3.52 | 44.37 | 13.38 | 38.73 | 142 | 75.68 | 12.56 |
| F059-jul2014-M1 | 17.76 | 44.74 | 11.84 | 25.66 | 152 | 88.61 | - |
| M066-apr2013-M1 | 22.34 | 14.18 | 16.31 | 47.16 | 282 | 71.45 | 4.59 |
| M066-jun2014-M2 | 20.24 | 40.48 | 5.95 | 33.33 | 84 | 100.65 | - |
| M069-jul2014-M1 | 19.15 | 31.91 | 12.06 | 36.88 | 141 | 70.34 | 0.26 |
| F070-jul2014-M1 | 7.32 | 36.59 | 11.85 | 44.25 | 287 | 62.48 | 3.431242503 |
| F071-jul2014-M2 | 17.75 | 20.63 | 25.33 | 36.29 | 383 | 49.92 | 13.95 |
| M074-jun2014-M2 | 11.21 | 48.71 | 19.83 | 20.26 | 232 | 85.04 | 47.3 |
| M075-jun2014-M1 | 27.66 | 17.87 | 24.26 | 30.21 | 235 | 76.68 | 25.57 |
| F076-jul2014-M1 | 2.76 | 22.58 | 35.02 | 39.63 | 217 | 104.58 | - |
| F081-jul2014-M1 | 15.7 | 19.73 | 25.11 | 39.46 | 223 | 59.97 | 13.56 |
| M086-jun2014-M2 | 7.04 | 13.38 | 34.51 | 45.07 | 142 | 55.79 | - |
| F100-jul2014-M2 | 12 | 43 | 8 | 37 | 100 | 94.17 | 0.15 |
| M103-jul2014-M1 | 2.48 | 72.73 | 0.83 | 23.97 | 121 | 122.45 | 4.56 |
| F106-jul2014-M1 | 1.71 | 49.66 | 18.15 | 30.48 | 292 | 61.95 | 25.49 |
| M111-apr2013-M1 | 16.18 | 19.09 | 15.77 | 48.96 | 241 | 53.84 | 1.55 |
