# Supplementary material for: Buccal dental-microwear and dietary ecology in a free-ranging population of mandrills (Mandrillus sphinx) from southern Gabon
Source: PLoS One. 2017 Oct 26;12(10):e0186870. doi: 10.1371/journal.pone.0186870 (PMC5658090; doi:10.1371/journal.pone.0186870)
Supplement: S3 Table — Eight qualitative scores were attributed to each analyzed SEM micrograph (degree of fuzziness or erosion, patina, presence of highly curved scratches, holes, artifacts or pits, presence of visible perikymata). A total of 48 LMM were run to study their individual effects on each of the six studied buccal microwear variables. These models included the main variables described in the main text (seasonality, individuals’ age and sex, tooth identity and tooth part). For clarity sake, we present only significant results. The fuzziness score ranges from 1 (image not fuzzy) to 3 (between 25 and 50% of the image is fuzzy); the erosion score ranges from 1 (image not eroded) to 3 (three and more patches of erosion or one large patch of erosion covering ~10% of the image); the presence of highly curved scratches (measured in different segments) ranges from 1 (no scratch is highly curved) to 3 (at least five scratches are highly curved). (DOCX) [file pone.0186870.s003.docx]

**S3 Supporting Information**

**S3 Table. Effects of image quality on buccal microwear variables.** Eight qualitative scores were attributed to each analyzed SEM micrograph (degree of fuzziness or erosion, patina, presence of highly curved scratches, holes, artifacts or pits, presence of visible perikymata). A total of 48 LMM were run to study their individual effects on each of the six studied buccal microwear variables. These models included the main variables described in the main text (seasonality, individuals’ age and sex, tooth identity and part). For clarity sake, we present only significant results.

The fuzziness score ranges from 1 (image not fuzzy) to 3 (between 25 and 50% of the image is fuzzy); the erosion score ranges from 1 (image not eroded) to 3 (three and more patches of erosion or one large patch of erosion covering ~10% of the image); the presence of highly curved scratches ranges from 1 (no scratch is highly curved) to 3 (at least five scratches are highly curved), highly curved scratches are measured in different segments.

| **Dependent variables** | **Explanatory variables** | **F** | **P-value** |
| --- | --- | --- | --- |
| **Total number of scratches** | Season | 1.75 | 0.20 |
|  | Sex | 1.68 | 0.20 |
|  | **Age** | **10.29** | **0.01** |
|  | Tooth | 1.55 | 0.24 |
|  | **Tooth part** | **6.07** | **0.03** |
|  | **Fuzziness score** | **5.49** | **0.02** |
| **Average scratch length** | **Season** | **9.75** | **0.01** |
|  | Sex | 0.10 | 0.75 |
|  | **Age** | **4.8** | **0.04** |
|  | Tooth | 0.27 | 0.77 |
|  | Tooth part | 2.80 | 0.11 |
|  | **Erosion score** | **5.66** | **0.01** |
| **% disto-mesial scratches** | **Season** | **4.98** | **0.04** |
|  | **Sex** | **7.38** | **0.01** |
|  | Age | 0.80 | 0.39 |
|  | Tooth | 0.17 | 0.84 |
|  | Tooth part | 0.41 | 0.53 |
|  | **Presence of highly curved scratches** | **5.97** | **0.01** |
