# Supplementary material for: Buccal dental-microwear and dietary ecology in a free-ranging population of mandrills (Mandrillus sphinx) from southern Gabon
Source: PLoS One. 2017 Oct 26;12(10):e0186870. doi: 10.1371/journal.pone.0186870 (PMC5658090; doi:10.1371/journal.pone.0186870)
Supplement: S4 Table — These individuals were used to analyze the relationships between PDE and diet (Spearman correlation tests). Date of dental molding, individual’s age, PDE, residuals of PDE (independent of age) and percentages (noted “%”) of food items consumed according to their physical properties are provided in this table. The physical properties tested in this study are encoded as hard, soft and tough food items (mechanical properties) and monocotyledonous plants, where this plant clade indicates, a priori, a high concentration in phytoliths (food abrasiveness). (DOCX) [file pone.0186870.s004.docx]

**S4 Supporting Information**

**S4 Table. Detailed information about individuals for which both feeding behavior and PDE data are available.** These individuals were used to analyze the relationships between PDE and diet (Spearman correlation tests). Date of dental molding, individual’s age, measured PDE, calculated residuals of PDE (independent of age) and percentages (noted “%”) of food items consumed according to their physical properties are provided in this table. The physical properties tested in this study are encoded as hard, soft and tough food items (mechanical properties) and monocotyledonous plants, where this plant clade indicates, *a priori*, a high concentration in phytoliths (food abrasiveness).

| Individual’s identity | Date of capture | Age | PDE | Residuals of PDE | % hard | % soft | % tough | % monocotyledons |
| --- | --- | --- | --- | --- | --- | --- | --- | --- |
| 2 | 25/04/2013 | 3.15 | 2.90 | 0.88 | 21.05 | 44.74 | 26.32 | 34.21 |
| 12 | 24/04/2013 | 8.48 | 16.81 | -11.49 | 51.72 | 31.03 | 13.79 | 3.45 |
| 13 | 24/04/2013 | 12.34 | 70.64 | 7.44 | 27.42 | 50.00 | 17.74 | 27.42 |
| 18 | 27/04/2013 | 4.65 | 5.48 | -0.14 | 45.45 | 45.45 | 6.06 | 30.30 |
| 23 | 24/04/2013 | 10.24 | 47.63 | 0.20 | 36.07 | 31.15 | 27.87 | 26.23 |
| 29 | 27/04/2013 | 14.21 | 79.55 | 10.12 | 24.18 | 50.55 | 21.98 | 34.07 |
| 30 | 28/04/2013 | 10.90 | 54.11 | 0.49 | 28.57 | 14.29 | 57.14 | 0.00 |
| 31 | 25/04/2013 | 7.65 | 33.41 | 11.70 | 17.86 | 53.57 | 19.64 | 39.29 |
| 33 | 27/04/2013 | 14.07 | 54.62 | -14.63 | 30.00 | 30.00 | 40.00 | 0.00 |
| 34 | 27/04/2013 | 4.06 | 2.47 | -1.39 | 30.77 | 55.77 | 11.54 | 38.46 |
| 42 | 29/04/2013 | 3.66 | 2.82 | -0.08 | 32.08 | 41.51 | 22.64 | 33.96 |
| 45 | 29/04/2013 | 12.48 | 73.36 | 9.45 | 26.79 | 37.50 | 33.93 | 36.84 |
| 48 | 25/04/2013 | 12.50 | 56.60 | -7.40 | 33.75 | 43.75 | 17.50 | 30.38 |
| 58 | 30/04/2013 | 6.08 | 8.73 | -3.03 | 23.53 | 33.33 | 41.18 | 32.00 |
| 66 | 29/04/2013 | 4.08 | 4.59 | 0.70 | 31.25 | 40.63 | 18.75 | 40.63 |
| 1 | 10/07/2014 | 3.36 | 4.01 | 1.41 | 22.41 | 44.83 | 27.59 | 36.84 |
| 2 | 01/07/2014 | 4.33 | 7.53 | 0.26 | 21.05 | 44.74 | 26.32 | 34.21 |
| 9 | 09/07/2014 | 5.85 | 13.19 | -1.96 | 47.06 | 41.18 | 11.76 | 29.41 |
| 12 | 02/07/2014 | 9.83 | 31.91 | -6.37 | 51.72 | 31.03 | 13.79 | 3.45 |
| 13 | 01/07/2014 | 13.52 | 68.43 | 2.93 | 27.42 | 50.00 | 17.74 | 27.42 |
| 18 | 03/07/2014 | 5.83 | 12.88 | -2.18 | 45.45 | 45.45 | 6.06 | 30.30 |
| 21 | 09/07/2014 | 15.16 | 90.18 | 16.69 | 29.63 | 45.68 | 18.52 | 44.44 |
| 23 | 01/07/2014 | 11.43 | 46.40 | -2.47 | 36.07 | 31.15 | 27.87 | 26.23 |
| 24 | 04/07/2014 | 2.59 | 0.78 | 1.60 | 34.38 | 34.38 | 28.13 | 34.38 |
| 25 | 04/07/2014 | 13.64 | 63.84 | -2.34 | 27.78 | 50.00 | 18.52 | 31.48 |
| 29 | 03/07/2014 | 15.40 | 77.49 | 3.14 | 24.18 | 50.55 | 21.98 | 34.07 |
| 31 | 09/07/2014 | 8.85 | 43.96 | 11.54 | 17.86 | 53.57 | 19.64 | 39.29 |
| 34 | 05/07/2014 | 5.26 | 11.85 | -0.17 | 30.77 | 55.77 | 11.54 | 38.46 |
| 38 | 02/07/2014 | 12.42 | 59.41 | 3.22 | 39.13 | 43.48 | 13.04 | 26.09 |
| 39 | 05/07/2014 | 6.84 | 37.24 | 16.58 | 25.81 | 46.77 | 24.19 | 32.26 |
| 40 | 09/07/2014 | 8.85 | 28.17 | -4.25 | 31.03 | 51.72 | 13.79 | 27.59 |
| 41 | 02/07/2014 | 6.83 | 14.96 | -5.65 | 17.24 | 46.55 | 31.03 | 29.31 |
| 43 | 01/07/2014 | 4.58 | 6.47 | -2.04 | 23.81 | 50.00 | 23.81 | 28.57 |
| 45 | 06/07/2014 | 13.66 | 78.62 | 12.27 | 26.79 | 37.50 | 33.93 | 36.84 |
| 48 | 01/07/2014 | 13.68 | 66.08 | -0.37 | 33.75 | 43.75 | 17.50 | 30.38 |
| 54 | 02/07/2014 | 17.83 | 72.37 | -6.77 | 27.55 | 35.71 | 30.61 | 19.39 |
| 56 | 08/07/2014 | 13.43 | 61.43 | -3.49 | 28.85 | 50.00 | 21.15 | 30.77 |
| 58 | 30/06/2014 | 7.25 | 12.56 | -10.46 | 23.53 | 33.33 | 41.18 | 32.00 |
| 69 | 05/07/2014 | 3.26 | 0.26 | -1.89 | 25.00 | 43.75 | 12.50 | 37.50 |
| 74 | 30/06/2014 | 13.25 | 47.30 | -16.20 | 34.31 | 38.24 | 27.45 | 21.57 |
| 75 | 30/06/2014 | 7.49 | 25.57 | 1.14 | 22.22 | 41.67 | 31.94 | 26.39 |
