# Supplementary material for: Buccal dental-microwear and dietary ecology in a free-ranging population of mandrills (Mandrillus sphinx) from southern Gabon
Source: PLoS One. 2017 Oct 26;12(10):e0186870. doi: 10.1371/journal.pone.0186870 (PMC5658090; doi:10.1371/journal.pone.0186870)
Supplement: S5 Table — For the sake of clarity, the mandrill population we studied is highlighted (*), by contrast with the other mandrill population, which corresponds to four samples collected in a museum (Estebaranz et al 2012). (DOCX) [file pone.0186870.s005.docx]

**S5 Supporting information**

**S5 Table.** **Comparison of buccal scratch patterns between the studied mandrills and other primates, considering average values for five microwear variables based on Estebaranz and colleagues (2012).** For the sake of clarity, the mandrill population we studied is highlighted (*), by contrast with the other mandrill population, which corresponds to 4 samples collected in a museum (Estebaranz et al 2012).

| **Species** | **Scratch density** | **% disto-mesial scratches** | **% mesio-distal scratches** | **% vertical scratches** | **% horizontal scratches** |
| --- | --- | --- | --- | --- | --- |
| ***Cercopithecus aethiops*** | 3.76 | 18.30 | 26.78 | 38.46 | 16.46 |
| ***Cercopithecus sp*** | 4.36 | 16.31 | 24.84 | 44.59 | 14.26 |
| ***Cercocebus torquatus*** | 4.45 | 33.82 | 26.74 | 18.32 | 21.12 |
| ***Colobus sp*** | 2.60 | 21.01 | 25.43 | 24.41 | 29.15 |
| ***Gorilla gorilla gorilla*** | 3.30 | 25.46 | 23.99 | 28.83 | 21.71 |
| ***Mandrillus sphinx**** | 5.62 | 37.33 | 14.45 | 27.55 | 20.55 |
| ***Mandrillus sphinx*** | 3.82 | 24.80 | 25.50 | 28.19 | 21.52 |
| ***Pan troglodytes troglodytes*** | 3.07 | 23.21 | 22.57 | 23.50 | 30.72 |
| ***Pan troglodytes verus*** | 2.51 | 29.85 | 35.63 | 14.31 | 20.20 |
| ***Papio anubis*** | 3.17 | 11.52 | 28.05 | 48.35 | 12.08 |

Reference :

Estebaranz F, Galbany J, Martinez LM, Turbòn D, Pérez-Pérez A. Buccal dental microwear analyses support greater specialization in consumption of hard foodstuffs for *Australopithecus anamensis*. J Anthropol Sci. 2012;90: 163–185. doi:10.4436/JASS.90006
